# COVID-19 Resulted in Lower Grades for Male High School Students and Students With ADHD

**DOI:** 10.1177/10870547211044211

**Published:** 2021-10-26

**Authors:** Rosanna Breaux, Nicholas C. Dunn, Joshua M. Langberg, Caroline N. Cusick, Melissa R. Dvorsky, Stephen P. Becker

**Affiliations:** 1 Virginia Polytechnic Institute and State University, Blacksburg, USA; 2 Virginia Commonwealth University, Richmond, USA; 3 Children’s National Hospital, Washington, DC, USA; 4 The George Washington University School of Medicine and Health Sciences, DC, USA; 5 Cincinnati Children’s Hospital Medical Center, OH, USA; 6 University of Cincinnati College of Medicine, OH, USA

**Keywords:** adolescence, ADHD, coronavirus, academic performance, remote learning

## Abstract

**Objective:**

Researchers have speculated that the COVID-19 pandemic may expand the academic performance gap experienced by at-risk students. We examined learning experiences during the 2020 to 2021 school year and the impact the pandemic has had on high school student grade point average (GPA), including predictors of change in GPA from 2019–2020 to 2020–2021.

**Method:**

Participants were 238 adolescents (55.5% male), 49.6% with attention-deficit/hyperactivity disorder (ADHD), in the United States. Adolescents reported on their GPAs via online surveys.

**Results:**

GPA significantly decreased on average from 2019–2020 to 2020–2021 school year. ADHD status and biological sex significantly moderated change—students with ADHD and male students reported decreased GPA, whereas students without ADHD and female students’ GPA did not change. Low income and Black/Latinx students had lower GPAs in both school years.

**Conclusion:**

It is imperative that additional supports be provided for at-risk students to help them catch up on missed learning during the pandemic.

With school closures and the impromptu transition to remote learning, the immediate response to the COVID-19 pandemic significantly disrupted learning starting in March 2020 in the United States. These disruptions continued during the 2020 to 2021 school year, as many students experienced multiple changes in learning format throughout the year (Lieberman, 2020). Researchers have speculated that the pandemic may exacerbate pre-existing academic difficulties and expand the academic performance gap for at-risk student populations (e.g., Reich et al., 2020). Populations that have been particularly highlighted as at-risk include students with pre-established learning difficulties, such as students with attention-deficit/hyperactivity disorder (ADHD), and students from lower socioeconomic statuses and racial minorities (see Reich et al., 2020).

Consistent with this speculation, research suggests that adolescents with ADHD are less likely to engage in remote learning and/or demonstrate more difficulties completing work remotely (Becker, Breaux et al., 2020), and to have more difficulty staying on task during remote learning (Roy et al., In Press). Additionally, parents of adolescents with ADHD had more difficulties supporting remote learning and less confidence in their ability to support learning relative to their peers (Becker, Breaux et al., 2020). Further, students with ADHD often require additional services to receive a comparable education to their peers (Masonbrink & Hurley, 2020; Van Lancker & Parolin, 2020), and research from the pandemic suggests limited continuation of school-based services during the pandemic (Becker, Breaux et al., 2020; McFayden et al., 2021). Likewise, early results from the pandemic suggest that students from low income and Black, Indigenuous, and Latinx families struggled more with the shift from in person to remote learning, with students from lower socioeconomic and ingenuous ethnic backgrounds being less likely to engage in remote learning (e.g., Asanov et al., 2021; Becker, Breaux et al., 2020).

There is also reason to believe that academic performance during the COVID-19 pandemic may differ based on biological sex. For example, research suggests that higher self-discipline gives female adolescents an academic edge resulting in higher academic performance across multiple core academic areas (English, Mathematics, and Social Studies) relative to male students (Duckworth & Seligman, 2006). Similarly, a meta-analysis conducted by Voyer and Voyer (2014) found females had higher academic achievement than males across all years of schooling (elementary, middle, and high school; university). Together, this research suggests that the COVID-19 pandemic may exacerbate the academic performance gap for males due to less self-discipline, something that is critical during remote learning.

Given this backdrop, the present study sought to better understand the disruptions to student learning during the 2020 to 2021 school year (learning format, change in learning format, and hours of direct instructions) and impact of the pandemic on homework performance and GPA. The primary aim of this study was to examine predictors of (ADHD status, biological sex, family income, race/ethnicity) change in high school student GPA from the 2019–2020 to 2020–2021 school years. This study is uniquely suited to assess this as our sample consists entirely of students who were in 11th and 12th grade during the 2020 to 2021 school year. These are years in which GPA is particularly salient as students plan to apply for college or enter the workforce post-graduation. Additionally, we sought to examine modifiable, academic-related predictors of change in GPA (i.e., academic amotivation, daily life executive function [EF] difficulties, difficulties with remote learning, and total hours of schoolwork during spring 2020) that may inform school supports and interventions for the upcoming 2021 to 2022 school year and beyond. These predictors were chosen as they are either highly salient difficulties among students with ADHD (i.e., amotivation, EF; Becker, 2020) or have been posited as potential areas for individual differences and risk during COVID-19-related remote learning (i.e., total hours of schoolwork, remote learning difficulties; Reich et al., 2020).

## Method

Participants were 238 adolescents (132 males; ages 15–17 years; *M* = 16.74, *SD* = 0.59) from two sites in the Southeastern and Midwestern United States. Adolescents were high school students in 10th and 11th grade during the 2019 to 2020 school year and 11th and 12th grade during the 2020 to 2021 school year. Approximately half of the sample (*n* = 118) was comprehensively diagnosed with ADHD prior to COVID-19 (see Becker et al., 2019; Langberg et al., 2019, for additional details). Adolescents identified as predominantly White (82%), with 7% identifying as biracial/multiracial, 6% identifying as Black, 4% Asian, and 1% identifying as another race; 4% of the sample identified as Latinx. Participants came from a range of socioeconomic backgrounds (*M* income = $93,073, *SD* = $34,856), with 19% of families falling below the 2019 United States median household income.

### Procedures

Participants who provided consent to be contacted for future research pre-COVID-19 (visits between September 2018 and February 2020; *N* = 262) were invited to participate in the current study, with COVID-19 data being collected over four time points: spring 2020 (May 15–June 14, 2020), summer 2020 (July 1–August 5, 2020), fall 2020 (October 1–November 15, 2020), and spring 2021 (March 1–May 15, 2021). In the current study, pre-COVID-19 data and data from the spring 2020, fall 2020, and spring 2021 time points are used. Importantly, the 238 participants who participated in the COVID-19 time points did not differ from the 24 participants who were contacted for possible participation on adolescent sex, race, ethnicity, ADHD symptoms, or family income (*p*s > .07). For more information on inclusion and exclusion criteria for the larger study, see Becker and colleagues (2019). All data were collected utilizing electronic surveys administered separately to parents and adolescents. Informed consent and assent were obtained, and parents and adolescents were compensated for their time completing questionnaires at each time point.

### Outcome Measures

#### GPA

In the fall 2020 survey, adolescents reported on their overall GPA for the 2019 to 2020 school year; values ranged from 1.5 to 4.0. Similarly, during the spring 2021 survey, adolescents reported on their overall GPA for the 2020 to 2021 school year; values ranged from 2.0 to 4.0. Since not all high schools enabled GPA to go above 4.0, values were capped at 4.0. Self-reported grades are a valid measure that is highly correlated with actual student grades (Sticca et al., 2017).

#### Remote learning experiences

The Home Adjustment to COVID-19 Scale (HACS; Becker, Quach et al., 2020) is a parent-report measure assessing experiencing with remote/hybrid learning, service use changes, and learning difficulties during the COVID-19 pandemic. The HACS was completed in spring 2020, fall 2020, and spring 2021. Composites used in this study include adolescent remote learning difficulties (range = 6–30). Item level data used in this study include learning format (fully in person, hybrid, fully remote), change in learning format, and hours of direct instruction/school day (range = 0–8 hours).

#### Homework performance

The Homework Performance Questionnaire (Power et al., 2007) was completed by parents pre-COVID-19 and at spring 2021 to assess the amount of time adolescents do a behavior consistently well (e.g., write down homework assignments independently, manage homework time well), with higher scores signifying better homework performance.

### Predictor Variables

#### ADHD status

During the initial in person pre-COVID-19 assessment, all participants underwent a comprehensive ADHD diagnostic evaluation. To be eligible for the ADHD group, adolescents were required to meet all Diagnostic and Statistical Manual for Mental Disorders, Fifth Edition criteria for either ADHD combined or predominantly inattentive presentation on the Children’s Interview for Psychiatric Syndromes (Weller et al., 2000) diagnostic interview and evidence impairment in home, academic, and/or social settings. Participants were included in the comparison group if parents endorsed <4 symptoms in both domains of ADHD (i.e., inattention, hyperactivity/impulsivity) on the diagnostic interview.

#### Demographic variables

Parents reported on adolescent biological sex (0 = *male*, 1 = *female*) and family income as part of a demographic questionnaire; a dichotomous variable was created based on the 2019 U.S. median family income of $68,703 (0 = below U.S. median, 1 = above U.S. median). Adolescents self-reported on their own racial and ethnic identities. Given that this sample was predominately non-Latinx and White, and the disproportionate impact the COVID-19 pandemic has had on Black and Latinx families, a dichotomous variable was created (0 = adolescent does not identify as Black or Latinx, 1 = adolescent identifies as Black or Latinx).

#### Academic amotivation

The amotivation subscale (e.g., “I can’t see why I go to school and frankly, I couldn’t care less”) of the Academic Motivation Scale (Vallerand et al., 1992) was assessed pre-COVID-19 to assess academic amotivation. Amotivation refers to an absence of intrinsic and extrinsic motivation, with amotivated students being often detached from their work or believing that effort will not impact outcomes.

#### Daily life EF difficulties

EF difficulties were assessed using parent-report pre-COVID-19 on the Behavior Rating Inventory of Executive Function – Second Edition (Gioia et al., 2002); the Global Executive Composite was used in the present study. Higher scores indicate more difficulties.

#### Remote learning experiences

Data from the HACS (Becker, Quach et al., 2020) was also used as a predictor of change in GPA. Specifically, the spring 2020 adolescent remote learning difficulties composite and total hours of school work item were predictors in the regression analysis.

## Results

### Learning Experiences during the 2020 to 2021 School Year

Only 4% of parents reported having a choice on the format of their child’s learning for the 2020 to 2021 school year. During fall 2020, 65.6% of parents reported a change in format during the first 2 months of school (e.g., changing from hybrid to fully remote), with only 9.7% of adolescents learning fully in person at the time of data collection; 38.8% were learning in a hybrid in person/remote format and 51.5% were learning fully remote. During spring 2021, 24.8% of parents reported a change in format during the first few months of the spring semester (i.e., since January 2021). However, the percentage of adolescents reported to be learning fully in person by spring 2021 increased to 38.3% (relative to 16.0% hybrid and 45.6% fully remote). Encouragingly, total hours of direct instruction the high school students were receiving significantly increased from spring 2020 (*M* = 1.65, *SD* = 1.52) to fall 2020 (*M* = 4.62, *SD* = 1.85), with hours of direct instruction significantly increasing again from fall 2020 to spring 2021 (*M* = 4.92, *SD* = 1.82), *F* = 195.81, *p* < .001. However, adolescent difficulties managing remote learning did not significantly improve from spring 2020 (*M* = 15.96, *SD* = 6.37) to either time point during the 2020 to 2021 school year (fall 2020: *M* = 15.90, *SD* = 6.09; spring 2021: *M* = 15.50, *SD* = 5.99), *F* = 0.54, *p* = .587. Notably, adolescent homework performance significantly decreased on average from pre-COVID-19 (*M* = 51.05, *SD* = 10.13) to during the 2020 to 2021 school year (*M* = 47.96, *SD* = 9.97), *t* = 4.35, *p* < .001, *d* = .32. Similarly, GPA significantly decreased on average from the 2019 to 2020 (*M* = 3.66, *SD* = 0.43) to 2020 to 2021 (*M* = 3.53, *SD* = 0.57) school year, *t* = 3.70, *p* < .001, *d* = .29.

### Predictors of Change in GPA from the 2019–2020 to 2020–2021 School Year

**Figure 1.**
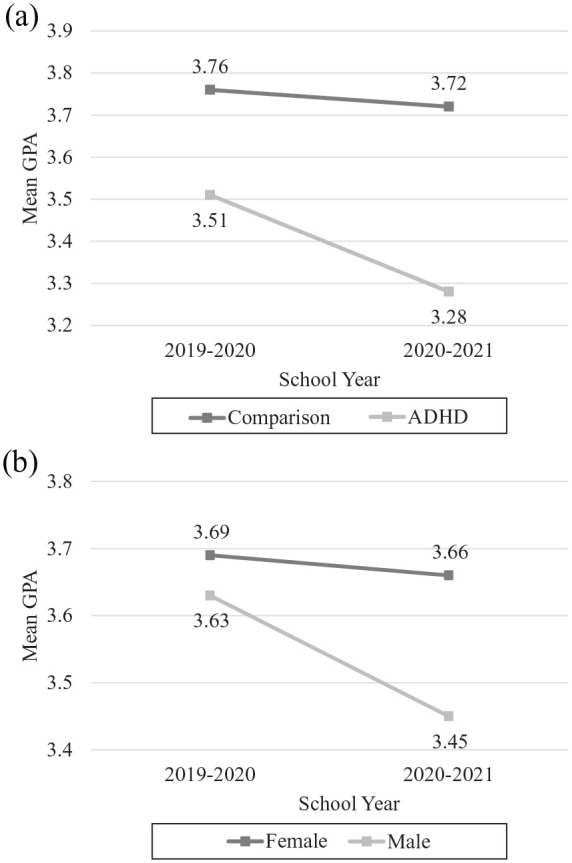
Changes in grade point average from the 2019–2020 to 2020–2021 School Year for (a) Adolescents with versus without ADHD and (b) females versus males. *Note.* GPA = grade point average.

The repeated measure ANOVA analysis indicated that ADHD status (*F* = 5.25, *p* = .023, partial η^2^ = .033) and biological sex (*F* = 3.97, *p* = .048, partial η^2^ = .025) significantly moderated change in GPA (see Table 1). Specifically, students with ADHD reported decreased GPA from the 2019–2020 to 2020–2021 school year, whereas students without ADHD had non-significant change in GPA (Figure 1a). Similarly, males experienced decreased GPA, whereas females experienced a non-significant change in GPA from the 2019–2020 to 2020–2021 school year (Figure 1b). Family income did not moderate change in GPA (*F* = 2.91, *p* = .090, partial η^2^ = .019); however, a main effect of income was found such that students with family incomes below the US median household income had lower GPAs during both school years on average, *F* = 12.11, *p* < .001, and GPA of students with incomes below and above the US median marginally or significantly decreased from 2019–2020 to 2020–2021 (see Table 1). Similarly, race/ethnicity did not moderate change in GPA (*F* = 1.80, *p* = .182, partial η^2^ = .012); despite the GPA of non-Black and/or Latinx students significantly decreasing and the GPA of Black and/or Latinx students remaining the same on average (see Table 1). However, a main effect of race/ethnicity was found such that Black/Latinx students had lower GPAs during both school years, *F* = 5.98, *p* = .016.

**Table 1.** Means and Standard Deviations for Grade Point Averages by Group Status.

	Spring 2020 GPA *M(SD)*	Fall 2020 GPA *M(SD)*	*t*	*p*	*d*
ADHD group	3.51 (0.53)	3.28 (0.64)	3.59	<.001	0.44
Comparison group	3.76 (0.29)	3.72 (0.42)	1.44	.154	0.15
Female students	3.69 (0.38)	3.66 (0.54)	0.71	.483	0.09
Male students	3.63 (0.45)	3.45 (0.57)	4.15	<.001	0.44
Students above U.S. median income	3.71 (0.34)	3.61 (0.49)	3.14	.002	0.27
Students below U.S. median income	3.37 (0.68)	3.13 (0.78)	1.98	.059	0.41
Black/Latinx students	3.26 (0.65)	3.27 (0.57)	−0.03	.974	−0.01
Non-Black/Latinx students	3.69 (0.38)	3.56 (0.56)	4.00	<.001	0.39

*Note.* U.S. median income is based on the 2019 median family income of $68,703. The *t*-statistic for the Full Sample is an independent samples *t*-test, all other *t*-statistics are from paired sample *t*-tests. GPA = grade point average.

To better understand factors that may have led to male students and students with ADHD experiencing decreases in GPA from 2019–2020 to 2020–2021, a multiple regression analysis was run. Potentially relevant predictors including, measures of pre-COVID-19 academic functioning (academic amotivation, EF difficulties) and measures of COVID-19 remote learning functioning during spring 2020 (total schoolwork hours, adolescent difficulties with remote learning) were examined as predictors of change in GPA. Pre-COVID-19 academic amotivation and daily life EF difficulties both emerged as predictors of change in GPA, such that students with greater academic amotivation and EF difficulties pre-COVID-19 were more likely to experience a decrease in GPA from 2019–2020 to 2020–2021 (see Table 2). Total hours of instruction during remote learning and remote learning difficulties did not significantly predict change in GPA.

**Table 2.** Predictors of Change in GPA from the 2019–2020 to 2020–2021 School Year.

	B	SE	β	p
Pre-COVID-19 academic amotivation	0.07	0.03	.18	.036
Pre-COVID-19 executive dysfunction	0.01	0.00	.22	.016
Spring 2020 total schoolwork hours	0.00	0.02	.00	.979
Spring 2020 remote learning difficulties	–0.01	0.01	–.09	.298

*Note.* Change in GPA = 2019–2020 GPA—2020–2021 GPA, such that positive values indicate a decrease in GPA and negative values indicate an increase in GPA. GPA = grade point average.

## Discussion

This study is the first to our knowledge to longitudinally examine the disruption in high school student learning caused by the COVID-19 pandemic from spring 2020 to spring 2021, and to examine changes in high school student homework performance and GPA during this time. Results support the hypothesis that the COVID-19 pandemic, and the disruptions to learning it caused, corresponded with significant drops in high school student homework performance and GPA. Further, findings suggest that the pandemic may have expanded the already present academic performance gap for high school students with ADHD, as well as male high school students relative to students without ADHD and female students (see Figure 1; Becker, 2020; Voyer & Voyer, 2014). Despite the pandemic not exacerbating the academic performance gap for low income and Black/Latinx students, these students displayed lower GPA on average during both school years.

Our finding of decreased GPA on average is consistent with other evidence from fall 2020 (Kuhfeld et al., 2020) suggesting smaller learning gains (particularly in math) from winter to fall 2020 relative to the prior school year. These findings are concerning, given evidence that adolescents are experiencing fewer learning difficulties during the pandemic relative to children (e.g., McFayden et al., 2021; Thorell et al., 2021), due to being better able to independently manage learning. This suggests that the academic impact may be even larger for younger students; this is an important area for future research. Regardless, it will be critical for all K-12 schools to provide opportunities for all students to catch up on missed learning from the COVID-19 pandemic (e.g., having the first month of the school year serve as a review from the prior year), and particularly for high schools to ensure that students graduate with the necessary knowledge prior to the transition to college or the work force.

Further, findings suggest that supports are particularly needed for male students and students with ADHD during the 2021–2022 school year to help them catch up on missed learning from the past year and a half. This is especially important given that male students and adolescents and young adults with ADHD are more likely to drop out of high school and not attend or finish college, relative to female students, and students without ADHD (e.g., Fried et al., 2016; Heckman & Lafontaine, 2010; Küpper et al., 2012). Our finding of decreased GPA for male students is alarming given early evidence that the COVID-19 pandemic has already resulted in a decline in college enrollment that is seven times as steep among men as women (Marcus, 2021). Although family income and race/ethnicity did not moderate change in GPA, a main effect was found for both, suggesting that adolescents from families below the US median household income and Black/Latinx students displayed lower GPA on average during both school years. This unfortunately is not surprising given extensive research suggesting that students from lower socioeconomic statuses and Black and Latinx students have lower academic achievement (see Gregory, 2000). However, since our sample was predominately non-Latinx/White and from a higher socioeconomic status (i.e., 9.2% identified as Black or Latinx and 18.6% had family incomes below the US median), we may have been underpowered to detect moderation based on these demographic factors and thus differential impact of the COVID-19 pandemic. Additional research with a greater range of diversity among students with regard to race, ethnicity, and socioeconomic status is desperately needed.

Finally, results from multiple regression analyses examining modifiable, academic predictors of change in GPA suggest that student amotivation and EF difficulties may be possible intervention targets to help reduce the academic impact of the COVID-19 pandemic upon the return to school during the 2021 to 2022 school year and beyond. Encouragingly, programs already exist at both the high school (e.g., Sibley, 2016) and college level (e.g., Anastopoulos et al., 2021; Meinzer et al., 2021) to support students with ADHD and enhance their academic motivation and EF abilities. Such group-based interventions could be critical in helping students with ADHD and male high school students and college freshman overcome the loss of learning caused by the COVID-19 pandemic, and are a critical area for future research.
